# Comparative Metabolomics Study of Four Kinds of Xihu Longjing Tea Based on Machine Fixing and Manual Fixing Methods

**DOI:** 10.3390/foods12244486

**Published:** 2023-12-14

**Authors:** Hongchun Cui, Yuxiao Mao, Yun Zhao, Haitao Huang, Junfeng Yin, Jizhong Yu, Jianyong Zhang

**Affiliations:** 1 Tea Research Institute, Hangzhou Academy of Agricultural Science, Hangzhou 310024, China; chc1134@126.com (H.C.); lhl5562@126.com (Y.M.); zxx531001@126.com (Y.Z.); hthuang309@hotmaill.com (H.H.); 2 Tea Research Institute, Chinese Academy of Agricultural Science, Hangzhou 310008, China; yinjf@tricaas.com

**Keywords:** Xihu Longjing tea, processing method, quality, comparative metabolomics, chemical composition

## Abstract

China Xihu Longjing tea is famous for its good flavor and quality. However, information on its related metabolites, except for flavonoids, is largely deficient. Different processing methods for China Xihu Longjing tea fixing—by machines at both the first and second step (A1), first step by machine and second step by hand (A2), first step by hand and second step by machine (A3), and by hand at both the first and second step (A4)—were compared using a UHPLC–QE–MS-based metabolomics approach. Liquid chromatography–mass spectrometry was used to analyze the metabolic profiles of the processed samples. A total of 490 metabolites (3 alkaloids, 3 anthracenes, 15 benzene and substituted derivatives, 2 benzopyrans, 13 coumarins and derivatives, 128 flavonoids, 4 furanoid lignans, 16 glycosides and derivatives, 5 indoles and derivatives, 18 isocoumarins and derivatives, 4 chalcones and dihydrochalcones, 4 naphthopyrans, 3 nucleosides, 78 organic acids and derivatives, 55 organooxygen compounds, 5 phenols, 109 prenol lipids, 3 saccharolipids, 3 steroids and steroid derivatives, and 17 tannins) were identified. The different metabolic profiles were distinguished using PCA and OPLS-DA. There were differences in the types and contents of the metabolites, especially flavonoids, furanoid lignans, glycosides and derivatives, organic acids and derivatives, and organooxygen compounds. There was a positive correlation between flavonoid metabolism and amino acid metabolism. However, there was a negative correlation between flavonoid metabolism and amino acid metabolism, which had the same trend as prenol lipid metabolism and tannins. This study provides new valuable information regarding differences in the metabolite profile of China Xihu Longjing tea processed based on machine fixing and on manual fixing methods.

## 1. Introduction

China Xihu Longjing tea is popular all over the world because of its unique flavor and quality. The traditional processing method of Xihu Longjing tea is stir-frying by hand, with the ten techniques of “Shaking, building, kneading, pressing, shaking, grabbing, pushing, bucking, pressing and grinding”, requires a high level of processing and “See tea do tea” levels [1]. The traditional method is not only inefficient, but also difficult to realize on scale and for the standardization of tea production [2]. With the development of industry, there has been a need for the optimization, standardization, and scaling of these processing methods, and with shortages in the labor force, machine frying has been introduced for the processing of Xihu Longjing tea. In recent years, the performance of flat-shaped tea frying machines as well as drum-type and reciprocating trough-type Longjing tea pot machines has been gradually improved, and the mechanized processing method for Xihu Longjing tea has been continuously enhanced.

At present, Xihu Longjing tea machine processing and manual processing coexist. Machine frying has greatly improved the production efficiency of Xihu Longjing tea, but the taste and aroma of the machine-fried tea are still inferior to the quality of tea made through traditional hand-frying. Gong et al. studied the effects of whole-hand crafting, machine–hand crafting, and whole-machine tea crafting on the quality of spring (high-grade) Longjing tea; in particular, the combination of machine and hand-crafting in the shaping of the product reaches even higher quality levels than the hand-frying of high-grade Longjing tea [3]. Shen et al. also compared the effects of all-hand crafting, machine–hand combination crafting, and all-machine crafting on the quality of high-grade Xihu Longjing tea [4]; the tea quality was affected by many factors, including the fresh leaf variety [5,6,7], green pot fixing method [8], and Hui pot fixing method [9,10,11,12,13], as well as the brewing conditions [14,15] and evaluation fixing methods [16,17]. The fixing is the key link in the formation of the flavor quality of Xihu Longjing tea; it is mainly stir-fried at high temperatures so as to make the grass gas in the raw material of the fresh leaves evaporate. The color and shape of the tea basically takes shape at this point, when the water content of the tea is reduced from 75% to 25–30%. Research on the difference in quality achieved in Xihu Longjing tea production based on the different machine processing and manual processing methods is relatively weak.

The raw materials and processing technology of the fresh tea leaves has a significant influence on the taste of Xihu Longjing tea [18]. Spring buds are usually selected as raw materials for Xihu Longjing tea processing—usually picked around March. The fresh leaves of tea tree plants are treated by the processing technology of killing, rolling, and drying, which promotes the conversion of fresh leaf chemicals into various flavor chemicals. The changes in the various flavor chemicals during the processing of Xihu Longjing tea have been studied by scientists [19]. There has been much research on the change trends of the total contents of tea polyphenols, catechins, free amino acids, and tea polysaccharides during the processing of Longjing tea from Xihu. However, this research has not been deep enough. The flavonoids, glycosides, organic acids, and anthracenes are different in their flavor characteristics, and have different effects on the flavor of Xihu Longjing tea [20,21]. At present, the main processing methods for Xihu Longjing tea are either machine processing or manual processing. Few studies have focused on the combination of machine and manual methods. The different processing sequences of machines and hand-crafting results in different changes in the flavor and quality of Xihu Longjing tea. Information on this is still relatively scarce. In this study, changes in the non-volatile components of Xihu Longjing tea during different processing sequences were analyzed.

The classic research methods for tea chemistry mainly rely on biochemical analysis, which can only analyze a few compounds and cannot understand changes in multiple metabolites in tea. Metabolomics technology is a new research method for the study of tea flavor chemistry. Metabolomics has been used to analyze the flavor chemistry of white tea and green tea [22,23]. Targeted metabolomics analysis or non-targeted metabolomics analysis has always been used, which have problems such as a narrow detection range, low sensitivity, and low accuracy. Widely targeted metabolomics could effectively avoid the drawbacks of non-targeted and targeted analysis, and the approach has high flux and sensitivity [21]. Therefore, widely targeted metabolomics was used in this study to compare non-volatile metabolites in Xihu Longjing tea undergoing different processing techniques.

To clarify the quality difference mechanism of Xihu Longjing tea based on different machine processing and manual processing methods, we employed a combination of manual and mechanical processing methods, obtaining Xihu Longjing tea of different qualities. The flavor compounds of Xihu Longjing tea processed using a combination of machine and manual techniques are detected via ultraperformance liquid chromatography MS/MS (UPLC-MS/MS). In addition, multiple statistical analysis methods, including principle component analysis (PCA), orthogonal partial least squares discrimination analysis (OPLS-DA), and heat maps are used to compare varying flavor compounds in each sample, thus clarifying the effect of different machine processing and manual processing methods on these metabolites. This study can provide reference for the study of processing technology control and the flavor quality formation of Xihu Longjing tea.

## 2. Materials and Methods

### 2.1. Materials

The raw materials, namely, tea leaves, were picked from Longjing tea plantation in Xihu district, Hangzhou City, Zhejiang province.

The methanol, acetonitrile, and ethanol (chromatographic grade) were obtained from Thermo (Waltham, MA, USA). All standard samples were obtained from Sigma-Aldrich (St. Louis, MO, USA). Anhydrous ethanol was obtained from Dingguo Co., Ltd. (Shanghai, China). L-2-chlorophenylalanine was obtained from Shanghai Hengchong Co., Ltd. (Shanghai, China). Deionized water was obtained from the Millipore system (Molsheim, France).

### 2.2. Xihu Longjing Tea Processing Method

(1) Method 1 involved the fixing of Xihu Longjing tea by machine during both the first and second steps (A1)

Fresh tea leaves → spreading out (leaf thickness 2 cm for 4 h at 18–23 °C with a relative air humidity of 58%) → first fixing by machine (210 °C for 4 min, 6CCB-100ZD fixing machine, Zhejiang Hongwuhuan Tea Equipment Co., Ltd. (Quzhou, Zhejiang, China)) → spreading to cool (30 min) → second fixing by machine (170 °C for 4 min, 6CCB-100ZD fixing machine, Zhejiang Hongwuhuan Tea Equipment Co., Ltd.) → spreading to cool (30 min) → dry (90 °C for 60 min, 6CLH-60 drying machine, Shangyang Machinery Co., Ltd. (Quzhou, Zhejiang, China)) → dried tea (A1).

(2) Method 2 involved fixing the Xihu Longjing tea fixing by machine in the first step and by hand in the second step (A2)

Fresh tea leaves → spreading out (leaf thickness 2 cm for 4 h at 18–23 °C with a relative air humidity of 58%) → first fixing by machine (210 °C for 4 min, 6CCB-100ZD fixing machine, Zhejiang Hongwuhuan Tea Equipment Co., Ltd. (Quzhou, Zhejiang, China)) → spreading to cool (30 min) → second fixing by hand (190 °C for 10 min) → spreading to cool (30 min) → dry (90 °C for 60 min, 6CLH-60 drying machine, Shangyang Machinery Co., Ltd. (Quzhou, Zhejiang, China)) → dried tea (A2).

(3) Method 3 involved fixing the Xihu Longjing tea by hand in the first step and by machine in the second step (A3)

Fresh tea leaves → spreading out (leaf thickness 2 cm for 4 h at 18–23 °C with a relative air humidity of 58%) → first fixing by hand (200 °C for 10 min) → spreading to cool (30 min) → second fixing by machine (170 °C for 4 min, 6CCB-100ZD fixing machine, Zhejiang Hongwuhuan Tea Equipment Co., Ltd. (Quzhou, Zhejiang, China)) → spreading to cool (30 min) → dry (90 °C for 60 min, 6CLH-60 drying machine, Shangyang Machinery Co., Ltd. (Quzhou, Zhejiang, China)) → dried tea (A3).

(4) Method 4 involved fixing the Xihu Longjing tea by hand in both the first and second steps (A4)

Fresh tea leaves → spreading out (leaf thickness 2 cm for 4 h at 18–23 °C with a relative air humidity of 58%) → first fixing by hand (200 °C for 10 min) → spreading to cool (30 min) → second fixing by hand (190 °C for 10 min) → spreading to cool (30 min) → dry (90 °C for 60 min, 6CLH-60 drying machine, Shangyang Machinery Co., Ltd. (Quzhou, Zhejiang, China)) → dried tea (A4).

Each method of processing the Xihu Longjing tea was repeated three times.

### 2.3. Method for Preparing Sample Solution

At this stage, 80 mg of an accurately weighed sample was transferred to a 1.5 mL Eppendorf tube. Two small steel balls were added to the tube. Then, 20 μL L-2-chlorophenylalanine (0.3 mg/mL) was dissolved in methanol as an internal standard. Following this, a 1 mL mixture of methanol and water (7/3, *v*/*v*) was added to each sample. The samples were placed at −20 °C for 2 min and ground at 60 Hz for 2 min. The whole samples were extracted through ultrasonication for 30 min in an ice-water bath and then placed at −20 °C for 20 min. The samples were centrifuged at 4 °C (10,000 rpm) for 10 min. The supernatant was passed through a 0.22 μm filter and transfer to an LC vial. The vials were stored at −80 °C until LC–MS analysis.

QC samples were prepared by mixing aliquots of all of the samples.

### 2.4. Waters Q-EXACTIVE Plus/Dionex U3000 UHPLC

An ACQUITY UPLC I-Class system (Waters Corporation, Milford, CT, USA) coupled with a Q-EXACTIVE mass spectrometer (Waters Corporation, Milford, CT, USA) was used to analyze the metabolic profiling in both ESI-positive and ESI-negative ion modes. An ACQUITY UPLC HSS T3 column (1.8 μm, 2.1 × 100 mm) was employed in both positive and negative modes. Water containing 0.1% formic acid was used as mobile phase A. Water and acetonitrile 2/3(*v*/*v*) containing 0.1% formic acid was used as mobile phase B. Linear elution conditions and mass spectrometric analysis methods were used [24].

The QCs were injected at regular intervals (every three samples) throughout the analytical run to provide a set of data from which repeatability could be assessed.

### 2.5. Data Preprocessing and Statistical Analysis

The original LC-MS data were processed by Progenesis QI V2.3 (Nonlinear, Dynamics, Newcastle, UK) for baseline filtering, peak identification, integral, retention time correction, peak alignment, and normalization. The main parameters of 5 ppm precursor tolerance, 10 ppm product tolerance, and 5% product ion threshold were applied. Compound identification was based on precise mass-to-charge ratio (*m*/*z*), secondary fragments, and isotopic distribution using the Human Metabolome Database (HMDB), Lipidmaps (V2.3), Metlin, EMDB, PMDB, and self-built databases to conduct the qualitative analysis.

The extracted data were further processed by removing any peaks with a missing value (ion intensity = 0) in more than 50% of the groups by replacing the zero value by half of the minimum value, and by screening according to the qualitative results of the compound. Compounds with resulting scores below 36 (out of 60) points were also deemed to be inaccurate and removed. A data matrix was combined from the positive and negative ion data.

The matrix was imported into R to carry out Principle Component Analysis (PCA) to observe the overall distribution among the samples and the stability of the whole analysis process. Orthogonal Partial Least Squares Discriminant Analysis (OPLS-DA) and Partial Least Squares Discriminant Analysis (PLS-DA) were utilized to distinguish the metabolites that differed between the groups. To prevent overfitting, sevenfold cross-validation and 200 Response Permutation Testing (RPT) were used to evaluate the quality of the model.

The Variable Importance of Projection (VIP) values obtained from the OPLS-DA model were used to rank the overall contribution of each variable to group discrimination. A two-tailed Student’s T-test was further used to verify whether the metabolites of difference between the groups were significant. Differential metabolites were selected with VIP values greater than 1.0 and *p*-values less than 0.05.

## 3. Results and Discussion

### 3.1. Metabolite Profiling Analysis

**Figure 1:**
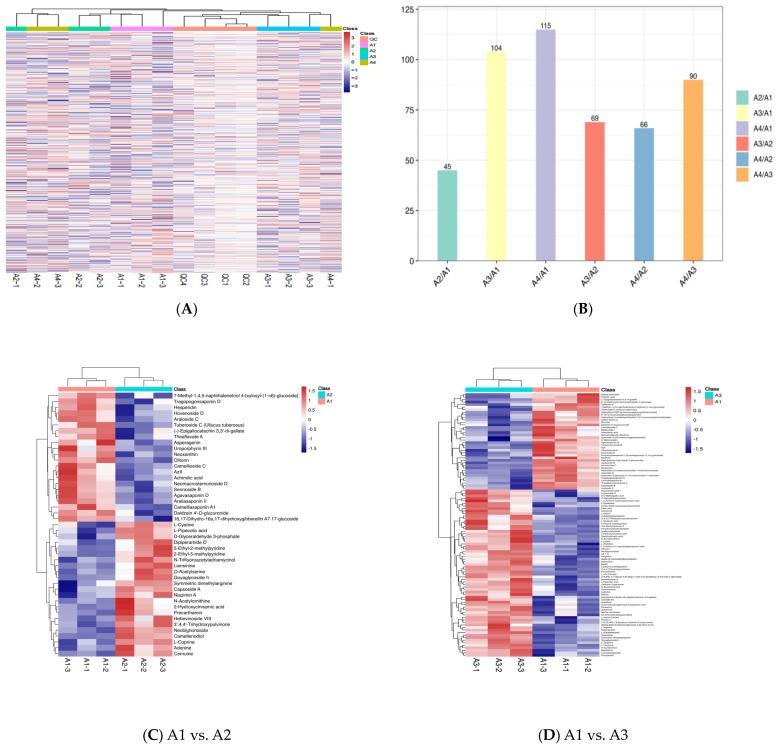

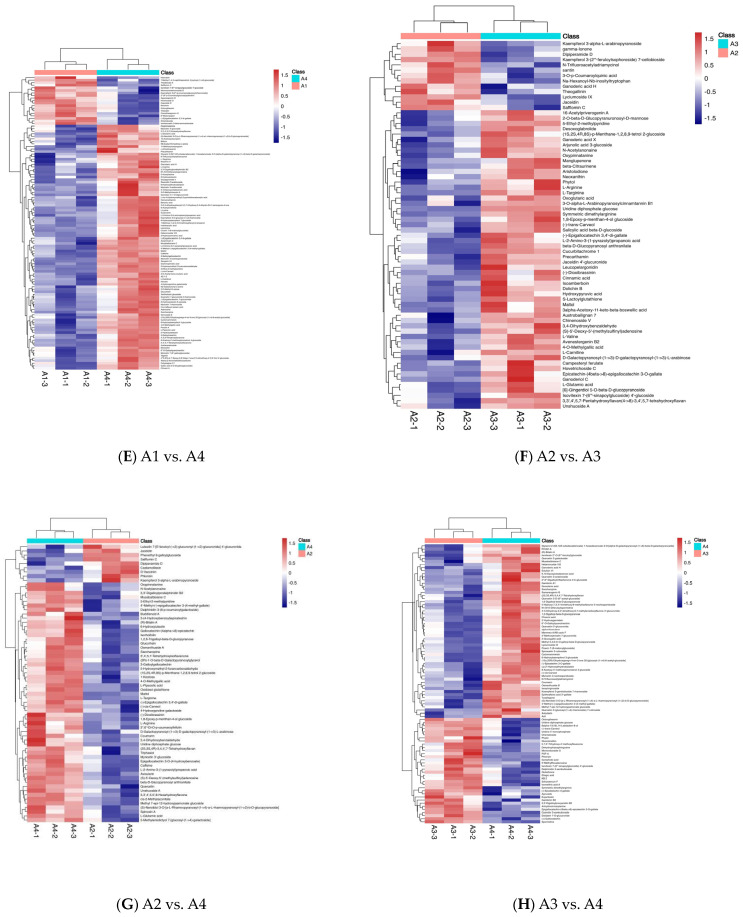
Heat map of the four Xihu Longjing tea samples. (**A**): heat map analysis of non-volatile components in four processes; (**B**): number of differential metabolites per two processes; (**C**): heat map analysis of non-volatile components in A1 vs. A2; (**D**): heat map analysis of non-volatile components in A1 vs. A3; (**E**): heat map analysis of non-volatile components in A1 vs. A4; (**F**): heat map analysis of non-volatile components in A2 vs. A3; (**G**): heat map analysis of non-volatile components in A2 vs. A4; (**H**): heat map analysis of non-volatile components in A3 vs. A4.

The non-volatile chemicals of four processing processes of Xihu Longjing tea were analyzed using UPLC-MS/MS. Extensive LC-MS/MS-based targeted metabolomics was used to analyze the differences in the metabolic profiles between the different treatments [21]. A total of 490 metabolites (3 alkaloids, 3 anthracenes, 15 benzene and substituted derivatives, 2 benzopyrans, 13 coumarins and derivatives, 128 flavonoids, 4 furanoid lignans, 16 glycosides and derivatives, 5 indoles and derivatives, 18 isocoumarins and derivatives, 4 chalcones and dihydrochalcones, 4 naphthopyrans, 3 nucleosides, 78 organic acids and derivatives, 55 organooxygen compounds, 5 phenols, 109 prenol lipids, 3 saccharolipids, 3 steroids and steroid derivatives, 17 tannins) were identified (Figure 1A). In order to view the specific differential metabolite quantity of each comparison group, the differential metabolite quantity of each comparison group was counted. The number of different metabolites were 45, 104, 115, 69, 66, and 90 in A1 and A2, A1 and A3, A1 and A4, A2 and A3, A2 and A4, and A3 and A4, respectively (Figure 1B).

The relative content of some metabolites above A1 was higher than that of A2, as shown in Figure 1C, such as for 7-methy-1,4,5-naphthalenetriod-4-[xylosyl-(1–6-glucoside], tragopogonsaponin D, hesperidin, hovenoside D, araloside, tuberoside C, (-)-epigallocatechin 3,3′-di-gallate, theaflavate A, asperagenin, uroporphyrin Ⅲ, neoxanthin, olitorin, camellioside C, azll, achimilic acid, neomacrostemonoside D, sennoside B, agavasaponin D, araliasasponin, araliasaponin Ⅱ, camelliasaponin A1, daddzen 4′-glucuronide, 16,17-dihydro-16a, and 17-dihydroxygibberellin A7 17-glucoside. However, the relative content of some metabolites below A1 was lower than that of A2, such as for L-cystine, L-pipecolic acid, D-glyceraldehyde 3-phosphate, d-ipiperamide D, 5-ethyl-2-methypyridine, 2-ethyl-5-methypyridine, N-trifluoroacetyladriamycinol, liensinine, O-acetylserine, goyaglycoside h, niazimin A, N-acetylornithine, 2-hydroxycinnamic acid, parcathamin, hebevinoside vill, 3′,4,4′-trihydroxypulvione, nebignoside, camellenodiol, L-coprine, adenine, and cernuine.

The relative content of some metabolites above A1 was higher than that of A3, as shown in Figure 1D, such as for rhoifolin, hovenoside D, hesperidin, ceposide D, kaempferol 3-arabinofuranoside 7-rhamnofuranoside, dibuthy phthalate, (-)-epigallocatechin 3,3′-di-gallate, 6″-4-hydroxycinnamoyl)astragalin 4′-glucoside, quercetin 3-(2G-rhamnosygentiobioside), kamempferol 3-[2″-(p-coumaroyglucosyl)rhamnoside], kaempferide 5-glucoside-7-glucuronide, biorobin, agvasaponin D, and Congmuyenoside B. However, the relative content of some metabolites below A1 was lower than that of A3. The relative content of some metabolites above A1 was higher than that of A4, as shown in Figure 1E, such as for kaempferol 3-[2″-(p-coumaroylglucosyl)rhamnoside], hovenoside D, ceposide D, theaflavate A, oolongtheanin, (-)-epigallocatechin-3,3′–di–gallate, and orientaloside. However, the relative content of some metabolites below A1 was lower than that of A4.

The relative content of some metabolites above A2 was higher than that of A3, as shown in Figure 1F, such as for dipiperamide D, kaempferol 3-(2‴-feruloylsophoroside) 7-cellobioside, N–Trifluoroacetyladriamycinol, lyciumoside IX, and 3-O-p-Coumaroylquinic acid. However, the relative content of some metabolites below A2 was lower than that of A3. The relative content of some metabolites above A2 was higher than that of A4 (Figure 1G), such as for luteolin 7–[E–feruloyl–(–>2)–glucuronyl–(1–>2)–glucuronide] 4′–glucuronide, phenethyl 6–galloylglucoside, dipiperamide D, castamollissin, and d–Vacciniin. However, the relative content of some metabolites below A2 was lower than that of A4.

The relative content of some metabolites above A3 was higher than that of A4, as shown in Figure 1H, such as for anhydrocinnzeylanine, epigallocatechin–(4beta–>8)–epicatechin 3-O-gallate, 3,3′–Digalloylprocyanidin B2, (-)-epiafzelechin 3-gallate, (+)–gallocatechin, oolongtheanin, (-)-trans-carveol, uridine diphosphate glucose, oridine 5′–monophosphate, orientaloside, dehydrophytosphingosine, pSF–A, delphinidin 3–sambubioside, glutathione, ellagic acid, schizotenuin F, isomelitric acid A, cyanidin 3-sambubioside, 5′-Methylthioadenosine, and isovitexin 7-(6‴-sinapoylglucoside) 4′-glucoside. However, the relative content of some metabolites below A3 was lower than that of A4.

According to the results of the comparison of total metabolites and two metabolites, there were differences in the types and contents of the metabolites, especially flavonoids, furanoid lignans, glycosides and derivatives, organic acids and derivatives, and organooxygen compounds.

### 3.2. Differential Metabolite Analysis Based on PCA

**Figure 2:**
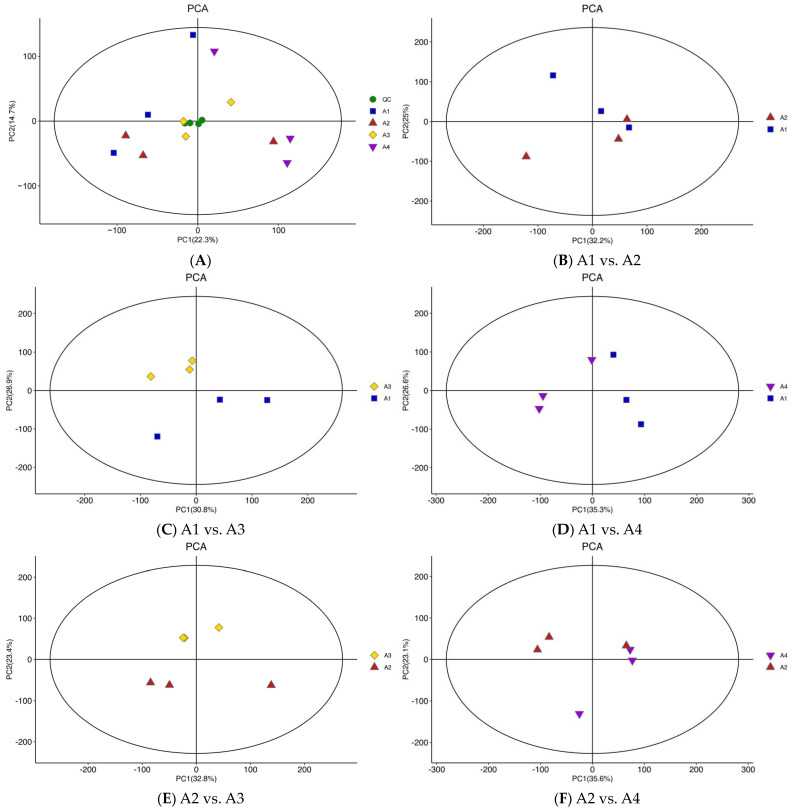

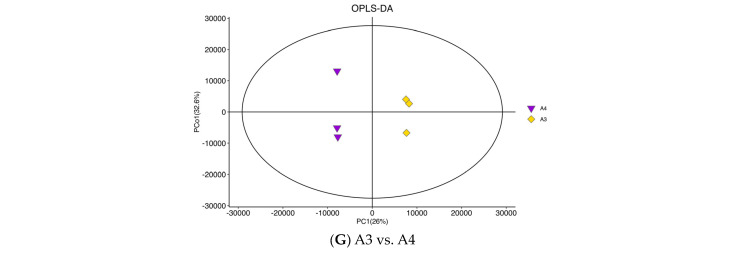
PCA of the relative differences in the metabolites in the four Xihu Longjing tea samples. (**A**): PCA of the relative differences in the metabolites from the four processes; (**B**): PCA of the relative differences in the metabolites in A1 vs. A2; (**C**): PCA of the relative differences in the metabolites in A1 vs. A3; (**D**): PCA of the relative differences in the metabolites in A1 vs. A4; (**E**): PCA of the relative differences in the metabolites in A2 vs. A3; (**F**): PCA of the relative differences in the metabolites in A2 vs. A4; (**G**): PCA of the relative differences in the metabolites in A3 vs. A4.

PCA is an unsupervised mode of data statistics that can restore the sample to the real state of metabolism. If there is an obvious trend of separation in the PCA score graph, it indicates that there is a significant difference in the composition of metabolites among the samples [25]. In the PCA plot of the four tea process method samples and the QC sample (Figure 2A), PC1 and PC2 are 22.3% and 14.7%, respectively. In the PCA plot comparing the two tea processing techniques (Figure 2B–G), the PC1 of A1 and A2, A1 and A3, A1 and A4, A2 and A3, A2 and A4, A3 and A4 is 32.2%, 30.8%, 35.3%, 32.8%, 35.6%, and 32.7%, respectively, and the PC2 of A1 and A2, A1 and A3, A1 and A4, A2 and A3, A2 and A4, A3 and A4 is 25%, 26.9%, 26.6%, 23.4%, 23.1%, and 23.6%, respectively. The small PC1 and PC2 values indicate that the degree of variation in these data is small.

Overall, the PCA plots of the four different tea processing samples indicate that significant differences in the metabolites can be proven. The different tea processing samples and QC can be distinguished from the A1, A2, A3, and A4 findings. The A1 sample overlaps with the A2 sample, the A2 sample overlaps with the A4 sample, and the A3 sample overlaps with the A4 sample, suggesting that a fraction of the metabolites in the A1 and A2 samples, A2 and A4 samples, and A3 and A4 samples are similar.

### 3.3. Differential Metabolite Analysis via OPLS-DA

**Figure 3:**
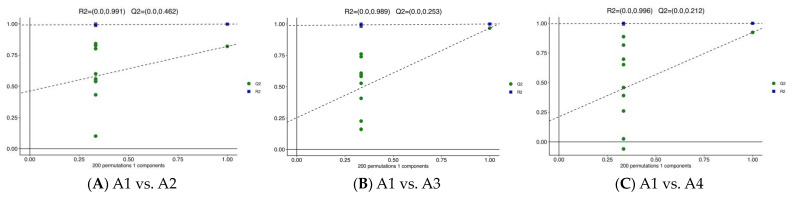

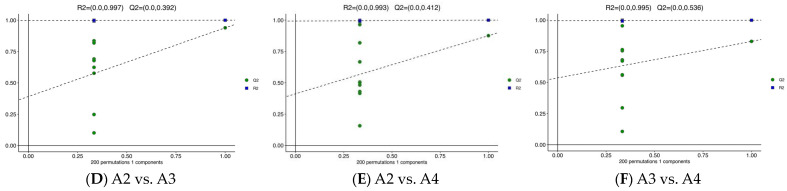
OPLS-DA of the relative differences in the metabolites in the four Xihu Longjing tea samples. (**A**): OPLS-DA models in A1 vs. A2; (**B**): OPLS-DA models in A1 vs. A3; (**C**): OPLS-DA models in A1 vs. A4; (**D**): OPLS-DA models in A2 vs. A3; (**E**): OPLS-DA models in A2 vs. A4; (**F**): OPLS-DA models in A3 vs. A4.

OPLS-DA is a supervised multivariate statistical analysis method that can effectively screen out differential metabolites [26]. OPLS-DA is a good method to identify the differential metabolites of Xihu Longjing tea made using different processes. The predictability (Q2) and goodness of fit (R2) of OPLS-DA models were observed for comparisons between A1 and A2 (Q2 = 0.462, R2 = 0.991, Figure 3A), as well as between A1 and A3 (Q2 = 0.253, R2 = 0.989, Figure 3B), A1 and A4 (Q2 = 0.212, R2 = 0.996, Figure 3C), A2 and A3 (Q2 = 0.392, R2 = 0.997, Figure 3D), A2 and A4 (Q2 = 0.412, R2 = 0.993, Figure 3E), and A3 and A4 (Q2 = 0.536, R2 = 0.995, Figure 3F). In the OPLS-DA models, the A1 and A2 samples, A2 and A4 samples, and A3 and A4 samples are clearly separated (Figure 3). The predictability (Q2) is good. The goodness of fit is strong.

**Figure 4:**
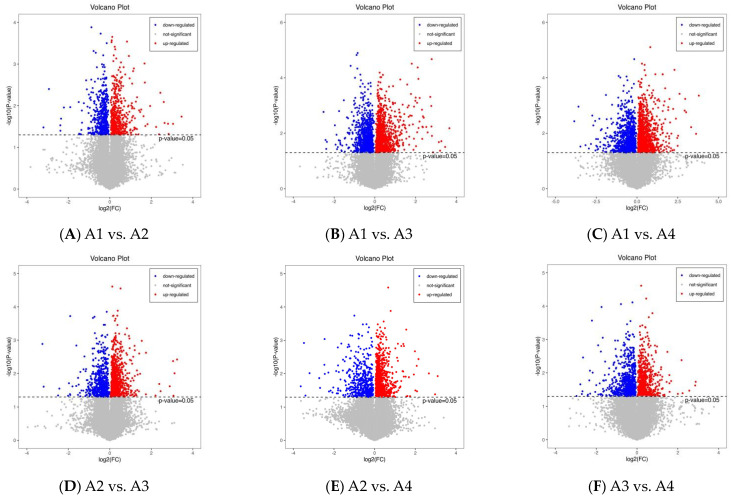
Volcano plots of the relative differences among the four Xihu Longjing tea samples. (**A**): volcano plots of up-regulated and down-regulated metabolites in A1 vs. A2; (**B**): volcano plots of up-regulated and down-regulated metabolites in A1 vs. A3; (**C**): volcano plots of up-regulated and down-regulated metabolites in A1 vs. A4; (**D**): volcano plots of up-regulated and down-regulated metabolites in A2 vs. A3; (**E**): volcano plots of up-regulated and down-regulated metabolites in A2 vs. A4; (**F**): volcano plots of up-regulated and down-regulated metabolites in A3 vs. A4.

**Figure 5:**
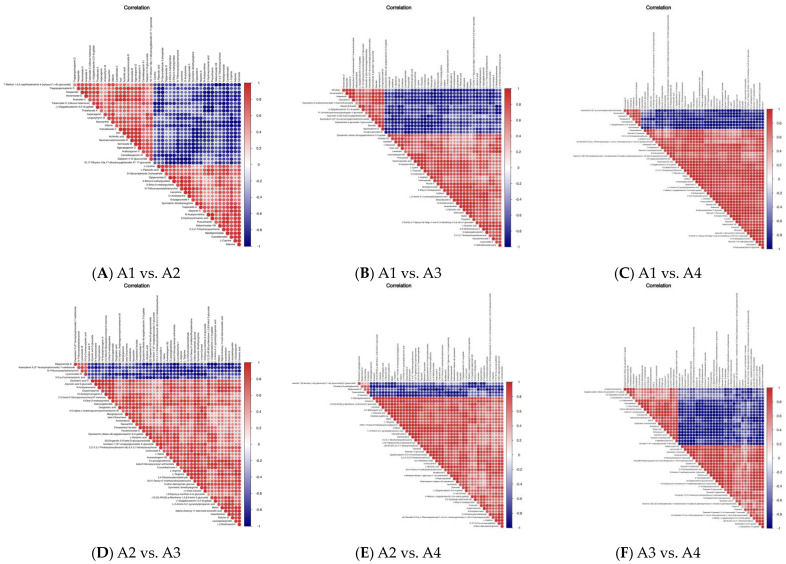
Differential metabolite association heatmap of the four Xihu Longjing tea samples. (**A**): correlation between individual metabolites in A1 vs. A2; (**B**): correlation between individual metabolites in A1 vs. A3; (**C**): correlation between individual metabolites in A1 vs. A4; (**D**): correlation between individual metabolites in A2 vs. A3; (**E**): correlation between individual metabolites in A2 vs. A4; (**F**): correlation between individual metabolites in A3 vs. A4.

Fold change and VIP score were used to screen for differential metabolites from 490 metabolites. *p* > 0.5 and VIP > 1.5 are the criteria for significant differences. The results are illustrated using volcano plots (Figure 4A–F). There are 401 differential metabolites (172 up-regulated and 229 down-regulated) between A1 and A2 (Figure 4A); 340 (up-regulated 204 and 136 down-regulated) between A1 and A3 (Figure 5B); and 261 (163 up-regulated and 98 down-regulated) between A1 and A4 (Figure 4C). Similarly, there are 319 (219 up-regulated and 100 down-regulated) between A2 and A3 (Figure 4D); 332 (237 up-regulated and 95 down-regulated) between A2 and A4 (Figure 4E), and 357 (204 up-regulated and 153 down-regulated) between A3 and A4 (Figure 4F).

### 3.4. Differential Metabolite Association Heatmap

The aim of differential metabolite association analysis is to examine the concordance of metabolites with the trend of metabolite change, and to analyze the correlation between individual metabolites by calculating the Plzeň correlation coefficient between all metabolites [27,28]. Metabolite correlations often reveal a synergy of changes between metabolites, such as a positive correlation for the same trend of change for a certain metabolite and a negative correlation for the opposite trend of change for another. The difference in the distribution of the red and blue colors in Figure 5 shows the synergy of changes between the metabolites in each comparison group. There was a positive correlation between flavonoid metabolism and amino acid metabolism. However, there was a negative correlation between flavonoid metabolism and amino acid metabolism (Figure 5). There was also a positive correlation between prenol lipid metabolism and tannin metabolism. However, there was a negative correlation between prenol lipid metabolism and tannin metabolism (Figure 5). Similarly, there was a positive correlation between prenol lipid metabolism and flavonoid metabolism. However, there was a negative correlation between prenol lipid metabolism and flavonoid metabolism (Figure 5).

### 3.5. Differential Metabolic Pathways among the Xihu Longjing Tea Samples

**Figure 6:**
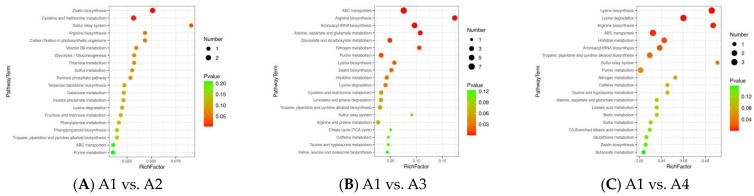

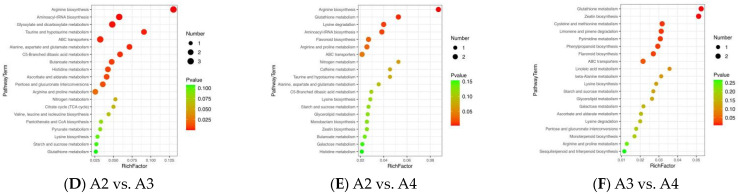
KEGG of the four Xihu Longjing tea samples. (**A**): metabolic pathways in A1 vs. A2; (**B**): metabolic pathways in A1 vs. A3; (**C**): metabolic pathways in A1 vs. A4; (**D**): metabolic pathways in A2 vs. A3; (**E**): metabolic pathways in A2 vs. A4; (**F**): metabolic pathways in A3 vs. A4.)

KEGG is a database for the systematic analysis of gene function and genome information that contains a lot of useful information [29]. The different metabolites of the four Xihu Longjing tea samples were mapped to the KEGG database (http://www.genome.jp/kegg/ (accessed on 8 June 2023)) to obtain detailed pathway information. The differential metabolites in the A1 and A2 samples were related to zeatin biosynthesis, L-Cysteine and methionine metabolism, and the sulfur relay system (Figure 6A). The differential metabolites in the A1 and A3 samples were related to ABC transporters, arginine biosynthesis, aminoacyl-tRNA biosynthesis, alanine, aspartate, and glutamate metabolism, nitrogen metabolism, and purine metabolism (Figure 6B). The differential metabolites in the A1 and A4 samples were related to lysine biosynthesis, lysine degradation, arginine biosynthesis, ABC transporters, histidine metabolism, aminoacyl-tRNA biosynthesis, tropane, piperidine, and pyridine alkaloid biosynthesis (Figure 6C). The results of the metabolic pathway analysis are as follows: 13/46, 28.26%, A1 vs. A2; 31/105, 29.52%, A1 vs. A3; 24/116, 20.69%, A1 vs. A4; 17/70, 24.29%, A2 vs. A3; 20/67, 29.85%, A2 vs. A4; 22/91, 24.18%, A3 vs. A4.

The differential metabolites in the A2 and A3 samples are related to arginine biosynthesis, aminoacyl-tRNA biosynthesis, glyoxylate and dicarboxylate metabolism, taurine and hypotaurine metabolism, ABC transporters, alanine, aspartate and glutamate metabolism, C5-Branched dibasic acid metabolism, butanoate metabolism, histidine metabolism, ascorbate and aldarate metabolism, and pentose and glucuronate interconversions (Figure 6D). The differential metabolites in the A2 and A4 samples are related to arginine biosynthesis, aminoacyl-tRNA biosynthesis, glyoxylate and dicarboxylate metabolism, taurine and hypotaurine metabolism, ABC transporters, alanine, aspartate and glutamate metabolism, C5-Branched dibasic acid metabolism, butanoate metabolism, histidine metabolism, ascorbate and aldarate metabolism, and pentose and glucuronate interconversions (Figure 6E). The differential metabolites in the A3 and A4 samples are related to arginine biosynthesis, glutathione metabolism, lysine degradation, aminoacyl-tRNA biosynthesis, flavonoid biosynthesis, arginine and proline metabolism, and ABC transporters (Figure 6F).

## 4. Conclusions

The quality of Xihu Longjing tea produced using different processing methods is quite different, especially when comparing tea processed by machine or by hand. Traditional Xihu Longjing is processed by hand, but at present, there are two ways to process the tea: by machine and by hand. However, there are few reports on the metabolite characterization and metabolic pathways involved in the machine and manual processing of Xihu Longjing tea, which limits the precision of quality control. In this study, metabolomics was used to analyze the metabolites in samples of Xihu Longjing tea processed using four different methods. A total of 490 metabolites were detected and identified in the Xihu Longjing tea samples. PCA and OPLS-DA were used to distinguish the different metabolism profiles of the four different Xihu Longjing tea processing methods. There was a significant difference in the composition of metabolites among the four Xihu Longjing tea samples. The PC1 and PC2 of the four tea process method samples and the QC sample were 22.3% and 14.7%, respectively. There were differences in the types and content of metabolites, especially flavonoids, furanoid lignans, glycosides and derivatives, organic acids and derivatives, and organooxygen compounds. There was a positive correlation between flavonoid metabolism and amino acid metabolism. However, there was a negative correlation between flavonoid metabolism and amino acid metabolism, which had the same trend as prenol lipid metabolism and tannins. This study provides new valuable information regarding the differences in the metabolite profile of China’s Xihu Longjing tea processed using machine fixing and manual fixing methods.

## Data Availability

Data are contained within the article.
